# Radioluminescence Response of Ce-, Cu-, and Gd-Doped Silica Glasses for Dosimetry of Pulsed Electron Beams

**DOI:** 10.3390/s21227523

**Published:** 2021-11-12

**Authors:** Daniel Söderström, Heikki Kettunen, Adriana Morana, Arto Javanainen, Youcef Ouerdane, Hicham El Hamzaoui, Bruno Capoen, Géraud Bouwmans, Mohamed Bouazaoui, Sylvain Girard

**Affiliations:** 1 Department of Physics, University of Jyväskylä, Survontie 9D, 40500 Jyväskylä, Finland; heikki.i.kettunen@jyu.fi (H.K.); arto.javanainen@jyu.fi (A.J.); 2 UJM, CNRS, IOGS, Laboratoire Hubert Curien, University of Lyon, UMR 5516, 18 rue Prof. B. Lauras, F-42000 Saint-Etienne, France; adriana.morana@univ-st-etienne.fr (A.M.); ouerdane@univ-st-etienne.fr (Y.O.); sylvain.girard@univ-st-etienne.fr (S.G.); 3 Department of Electrical and Computer Engineering, Vanderbilt University, Nashville, TN 37235, USA; 4 Univ-Lille, CNRS, UMR 8523-PhLAM-Physique des Lasers Atomes et Molécules, F-59000 Lille, France; bruno.capoen@univ-lille.fr (B.C.); geraud.bouwmans@univ-lille.fr (G.B.); mohamed.bouazaoui@univ-lille.fr (M.B.)

**Keywords:** dosimetry, electron accelerator, optical fiber, point dosimeter, pulsed electron beam, radiation-induced luminescence

## Abstract

Radiation-induced emission of doped sol-gel silica glass samples was investigated under a pulsed 20-MeV electron beam. The studied samples were drawn rods doped with cerium, copper, or gadolinium ions, which were connected to multimode pure-silica core fibers to transport the induced luminescence from the irradiation area to a signal readout system. The luminescence pulses in the samples induced by the electron bunches were studied as a function of deposited dose per electron bunch. All the investigated samples were found to have a linear response in terms of luminescence as a function of electron bunch sizes between $10^{-5}$ Gy/bunch and $1.5\times 10^{-2}$ Gy/bunch. The presented results show that these types of doped silica rods can be used for monitoring a pulsed electron beam, as well as to evaluate the dose deposited by the individual electron bunches. The electron accelerator used in the experiment was a medical type used for radiation therapy treatments, and these silica rod samples show high potential for dosimetry in radiotherapy contexts.

## 1. Introduction

A large part of the previous studies on radiation-induced luminescence (RIL) of doped silica glasses for ionizing radiation dosimetry has been done under X-ray irradiation. Regarding particle beams, most of the studies focused on proton beams. Examples of such studies are reported in [1], where Ce${}^{3+}$- and Cu${}^{+}$-doped samples were evaluated for proton therapy dosimetry purposes, and in [2], where Gd${}^{3+}$-doped silica glass was also studied. In these studies, a dose rate range of about 0.02–0.30 Gy/s from a continuous beam was used, and proton energies between 8 and 63 MeV were investigated. In [2], dose-depth profiles were also studied using Gd-, Cu-, and Ce-doped samples. A further review of optical fibre-based dosimetry for radiotherapy is reported in [3].

Ce-doped silica glass has been studied under X-ray irradiation in e.g., [4], where the optically stimulated luminescence (OSL) and RIL properties of the sample were investigated, and a linear RIL output for continuous dose rates between at least 26 and 1187 mGy/s was found. The electronic transition 4f–5d in Ce${}^{3+}$ ions is the basis for RIL emission, which is discussed in e.g., [4,5,6].

Ce-activated silica glass was also studied in [5]. The doped glass was tested with a continuous X-ray beam up to a dose rate of 50 Gy/s, with a linear output up to 30 Gy/s. At dose rates higher than 30 Gy/s, a luminescence response over the linear trend was observed.

Silica glass doped with Gd${}^{3+}$-ions has been studied in [7], where the RIL response under steady-state X-ray irradiation was found to be linear between at least 125 μGy(SiO${}_{2}$)/s and 12.25 Gy(SiO${}_{2}$)/s. The RIL of Gd${}^{3+}$ ions is ascribed to the transition between the ${}^{6}$P${}_{7/2}$ and ${}^{8}$S${}_{7/2}$ levels [2,7,8].

Cu-doped silica samples were studied in e.g., [9], in the shape of a photonic crystal fibre (PCF) under UV light excitation, and in [10], under X-ray irradiation. In [10], a linear trend of the luminescence response was also reported up to a dose rate of 30 Gy/s, and then a response over the linear trend above 30 Gy/s, as was the case for Ce-doped samples in [5]. In Cu${}^{+}$ ions, the transitions responsible for the RL emission are those from the state 3d${}^{9}$4s to the ground state 3d${}^{10}$ [9,11].

Very limited results of doped silica glass RIL responses to pulsed electron beams exist in the literature. In [12], a scintillating material (terbium-activated gadolinium oxysulfide) was placed in contact with a light-guiding fibre. The sample was then subjected to a beam of pulsed X-rays from a clinical linear accelerator (Clinac^®^). Studies of electron beams include those of thermoluminescence (TL) of Ge-doped optical fibers, such as in [13,14], and the same type of samples has been tested with other particles in e.g., [15]. The scintillation and OSL response of a Cu${}^{+}$-doped quartz glass was investigated in electron and X-ray beams from a Clinac in [16,17], where the sample was used to measure the total dose deposited during radiation runs.

In this paper, the RIL responses of Ce-, Cu-, and Gd-doped sol-gel silica glasses under a pulsed electron beam are investigated. The emission properties, and possibilities of monitoring the beam pulse-by-pulse with these samples are presented.

The interest and possibility of using these types of samples for dosimetry in the context of radiation therapy [1,2] makes the investigation of their responses to pulsed electron beams highly relevant. The particle accelerator used for irradiation tests in this study is a Clinac, and a characterization of the doped sol-gel silica rods in the pulsed Clinac electron beam opens the prospect of using the doped rods for dosimetry in a wider range of radiation therapy contexts.

## 2. Materials and Methods

### 2.1. Tested Samples

The tested materials were sol-gel glass rods. Further information regarding the production and fabrication of these samples can be found in previous publications, e.g., in [18,19]. Each sample, consisting of a rod drawn from doped silica glass, was fusion-spliced to 500-μm core multimode pure-silica core optical fibers (here referred to as transport fibers) to guide the induced RIL to the read-out electronics. The radioluminescent rods were approximately 1 cm long and 0.5 mm thick. Information about the tested samples and their doping concentrations are listed in Table 1.

### 2.2. Test Setup

At the end of the transport fiber, the signal readout system was located. For the tests of the response of the fibers as a function of electron pulse size, the readout system consisted of a photomultiplier tube (PMT) to convert the incident light to a voltage pulse, which was collected in an oscilloscope with high input impedance (1 MΩ).

**Figure 1:**
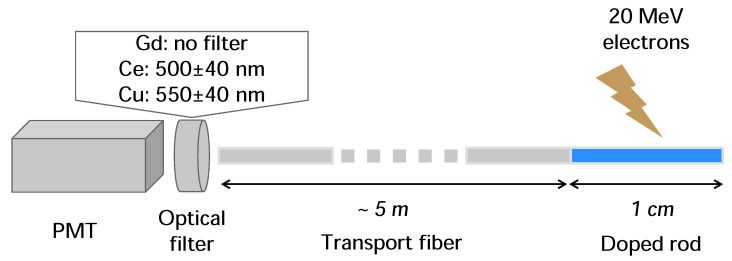
Schematic overview of the setup, where radiation-induced luminescence (RIL) in the doped rods are transported to a photomultiplier tube (PMT) through an optical filter.

The PMT, a Hamamatsu H9305-13 [20], was encased in a dark metal casing where the luminescent light from the sample could be collected from the transport fiber in the PMT window without background light contamination. A schematic of the PMT setup and a sample is shown in Figure 1, where the whole system was kept in darkness to shut out parasitic light. The transport fibers were running through a black tube, and the samples were covered with dark tape. Between the transport fiber and the PMT, an optical band-pass filter was placed to select a relevant wavelength span that included the RIL. The used filter was thus specific for each sample.

To measure the optical emission spectra from the samples, an Ocean Optics USB2000+ UV-VIS-ER spectrometer [21] was used. The transport fiber was then placed directly against the spectrometer window, which thus replaces the optical filter and PMT in Figure 1.

### 2.3. Test Methodology

To investigate the RIL response of the samples when subjected to a pulsed beam, a large number of RIL pulses were collected at a fixed beam setting. Then, the properties of the collected pulses in the oscilloscope were investigated after irradiation. The experimental procedure was the following for the different samples:

1 Fix a constant electron bunch size and frequency in the accelerator;
2 Start irradiation and keep on for circa 30 s, by irradiating until a fixed dose value;
3 Save the collected trace from the oscilloscope containing RIL pulses from the 30 s of irradiation;
4 Tune to a different electron bunch size and repeat.

During irradiation, a Si-diode detector was also located in the beam. The signal from this detector was saved as well, and used to identify electron bunches also in certain beam configurations where the signal in the tested sample was small. Such configurations consist, for example, in a shielded sample, in a sample positioned outside of the beam, or for very small bunch sizes.

**Figure 2:**
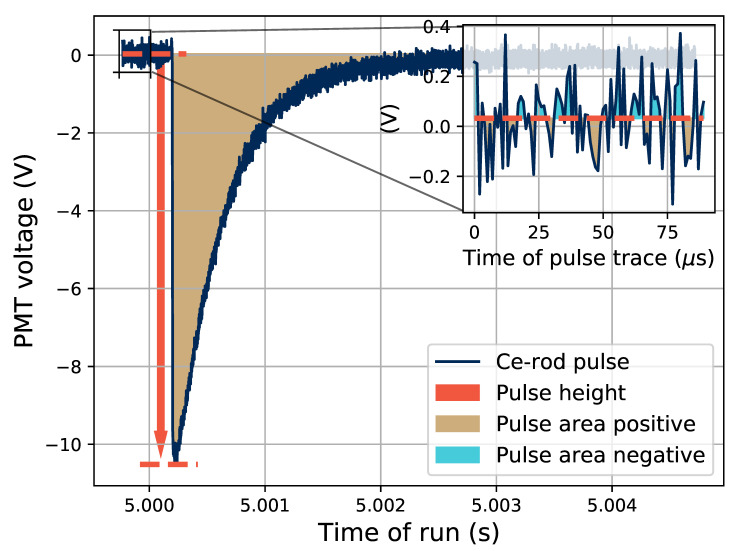
Example of one recorded pulse from a test using the Ce-rod, where the methods of calculating the height and area of the pulses are presented.

The saved traces of pulses were analyzed post-irradiation in terms of the height and area of the separate pulses. One pulse from a run with the Ce-rod sample is shown in Figure 2, where the separate procedures for determining the height and area of the pulses are presented. The baseline for the pulse was calculated as the average signal level immediately before the pulse, and the height of the pulse was recorded as the absolute difference between the pulse maximum and the calculated baseline, as shown in orange in the figure. The area was calculated as the absolute value of the integral of the pulse with respect to the calculated baseline, so that effectively the pulse area below the zero level was counted as positive, and the area above the zero level as negative.

### 2.4. Irradiation Facility, RADEF

The irradiation experiments presented in this paper were performed at the radiation effects facility (RADEF) at the accelerator laboratory of the University of Jyväskylä, Finland. A Varian Clinac 2100C/D [22] was used to generate the electron beam that was used in the experiments. At the facility, 6, 9, 12, 16, and 20 MeV electrons are available, with dose rates between 1 and 10 Gy(H${}_{2}$O)/min in standard operation. The dose rates mentioned here correspond to the dose rate at maximum dose depth in water. The machine was, however, not utilized in the standard mode of operation during the experiments presented in this paper, but instead used in a manner allowing for manual tuning of the amount of electrons present in the separate electron bunches from the machine.

**Figure 3:**
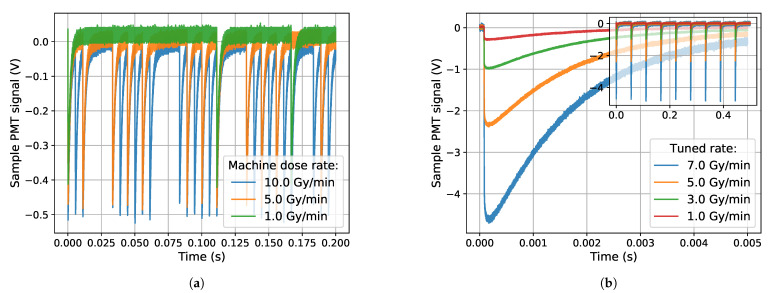
Dose rate tuning of the electron machine in different operating modes, recorded using a Cu-doped rod. (**a**) The standard dose rate tuning scheme is shown in the figure, where the bunch frequency is automatically modulated while the bunch sizes are kept constant. (**b**) The operation mode used in the experiments, where the bunch frequency is kept constant (shown in the figure inset) while the electron bunch size is tuned (see the varying size of the PMT pulses).

In the standard mode of operation (which was not utilized here), 5-μs long electron bunches are delivered at a frequency of up to 200 Hz when the machine is set to a dose rate of 10 Gy(H${}_{2}$O)/min. When the dose rate is lowered, an increasing number of 5-μs electron bunches are removed, so that when, e.g., running at 1 Gy(H${}_{2}$O)/min, 10 times fewer bunches are present than in the 10 Gy(H${}_{2}$O)/min operation, but the sizes of the individual electron bunches stay the same. This is shown for a few dose rate settings in Figure 3a, and is also discussed in e.g., [12]. In Figure 3, the PMT signal from consecutive electron bunches collected in an oscilloscope are shown (see Section 2.2) for different operating modes and different dose rates. The figures are made using the signal from a Cu-doped rod.

In this work, 20-MeV electrons were used at different dose rates, where the dose rate was modulated in a different manner than described above. Here the automatic dose rate regulation of the machine was turned off, and a certain bunch frequency was fixed. The bunch frequencies that were used were 20 and 200 Hz, corresponding to electron bunches delivered every 50 ms and 5 ms. Then the sizes of the electron bunches at the fixed frequency could be manually tuned by changing the current to the electron gun. This way, the responses of the samples to different sizes of electron bunches could be investigated. The dose rate tuning in this operating mode is shown in Figure 3b.

**Figure 4:**
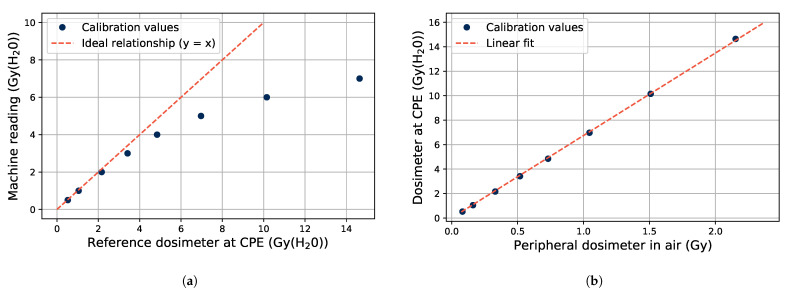
Response of the built-in dosimetry of the accelerator and external dosimeters when the electron bunch sizes are changed. The data points are each taken at a fixed dose rate (bunch size) for one minute of irradiation at a bunch frequency of 20 Hz. At this setting, the bunch size corresponding to the nominal machine value is 1 Gy/min. (**a**) Accelerator internal dosimetry against an external dosimeter at maximum dose depth in water. (**b**) Dosimeter at maximum dose depth in water against a peripheral dosimeter in air.

Changing the electron bunch sizes, so they become larger than normal, affects the functionality of the built-in dosimetry system of the electron accelerator. In the accelerator, there are ionization chambers which monitor the outgoing accelerator beam, which are meant to handle electron bunches of a certain size as shown in Figure 3a. As the bunches get larger, non-linearities in the built-in dosimeters are observed. This can be seen in Figure 4, where the nominal bunch size of the machine corresponds to a dose rate of 1 Gy(H${}_{2}$O)/min.

The saturation of the Clinacs internal ionization chambers at large bunch sizes is shown in Figure 4a, comparing the dose recorded by the machine with an external dosimeter (IBA PPC40 dosimeter [23]) at a maximum dose depth in water. Comparing this external dosimeter with a second one (IBA FC65-P [23]) located in the beam periphery in air, results in a linear relationship as seen in Figure 4b. The external dosimeters were used to ensure that the correct values of dose and dose rate were recorded, and they were used as the reference dosimeters during the experiments in the tests where electron bunches larger than nominally was used.

The values of dose and dose per electron bunch that are reported in this study refers to the electron fluence, which corresponds to said dose at maximum dose depth in water, and not the absorbed dose in the tested samples. During irradiation, the samples were located under a thin layer of darkening material (a black plastic sheet and a layer of black tape), but it can be approximated as the samples being located in air and being subjected to the immediate electron beam.

## 3. Results and Discussion

### 3.1. Emission Spectra of the Samples

**Figure 5:**
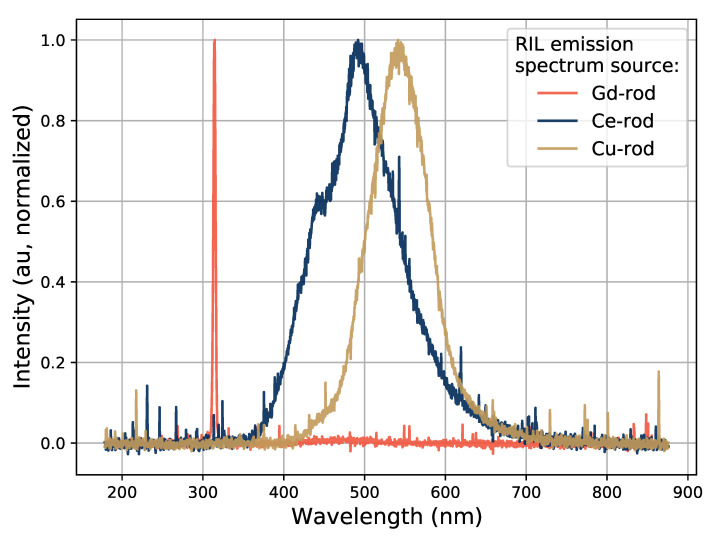
RIL emission spectra taken from the different sample types.

The measured RIL emission spectra of the samples are shown in Figure 5 for the different types of samples under 20-MeV electron irradiation. The emission spectra for the three different dopants correspond well to previously reported RIL emission spectra in the literature where X-rays were used as the excitation source. The reported spectra are all dominated by the expected RIL wavelengths without visible contamination from other sources such as Čerenkov radiation. No optical filters were used while obtaining the spectra presented in Figure 5.

The emission spectrum of the Gd-doped sample is a narrow peak at 314 nm. This is the same result as was discussed in [7], where a narrow emission peak at 314 nm was found under both X-ray and 275-nm UV excitation. The emission spectra of a Cu-doped sample under X-ray and 325-nm UV excitation was compared in e.g., [24], where the UV excited spectra was seen to be slightly broadened. This was ascribed to an increased emission from non-bridging oxygen hole centers (NBOHC) in the UV excitation case. The corresponding spectrum in Figure 5 peaks at 543 nm, and does not show this broadening. It is similar to the reported X-ray excited spectrum in [24].

This same comparison was done for a Ce-doped sample in [4] between X-ray and a 351-nm UV excited emission spectra. The X-ray emission spectrum in that study corresponds well to the one in Figure 5, however the knee structure around 450 nm is slightly less pronounced in [4] than it is here. Such differences can however be masked or amplified depending on the total transfer function of the detection system that was used (the combination of transport fiber and spectrometer), on calibrations of the spectrometer, and on potential post-processing of the data.

For the following tests, optical band-pass filters at 500±40 nm and 550±40 nm were used for the Ce-rod and the Cu-rod respectively, in front of the PMT window. No optical band-pass filter was used for the Gd-rod tests, since none were available that could cover the 314-nm emission peak of Gd.

### 3.2. Sample Response to Varying Electron Pulse Sizes

#### 3.2.1. Variations of Output Pulse Height

**Figure 6:**
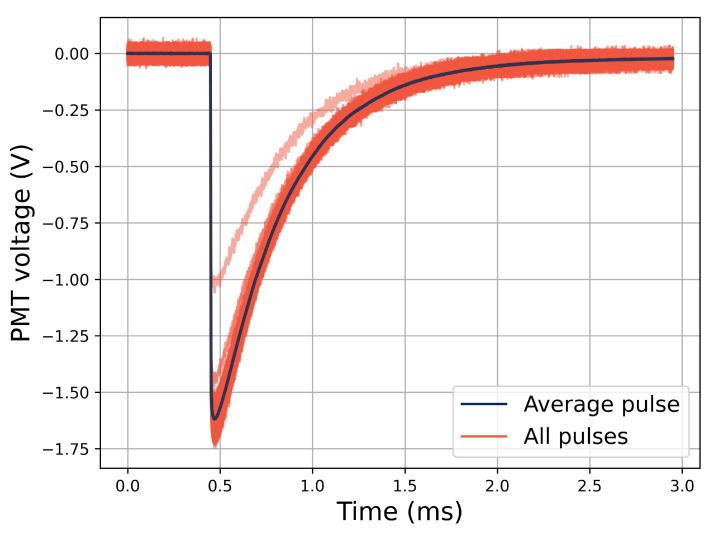
Collected pulses in the oscilloscope from 20-Hz electron bunch irradiation of the Ce-doped rod sample at $9.7\times 10^{-4}$ Gy/bunch.

Examples of collected pulses in the oscilloscope are shown in Figure 6. All the resulting pulses from the PMT for half a minute of irradiation at a fixed electron bunch frequency of 20 Hz, and at constant electron bunch sizes of $9.7\times 10^{-4}$ Gy/bunch impinging on the Ce-doped sample are displayed in the figure. The electron bunch that resulted in the pulse at 1.0 V in pulse height was the first recorded bunch of the run. This is a common behavior among all the runs in that the machine reaches the set bunch size after one or two smaller initial bunches.

**Figure 7:**
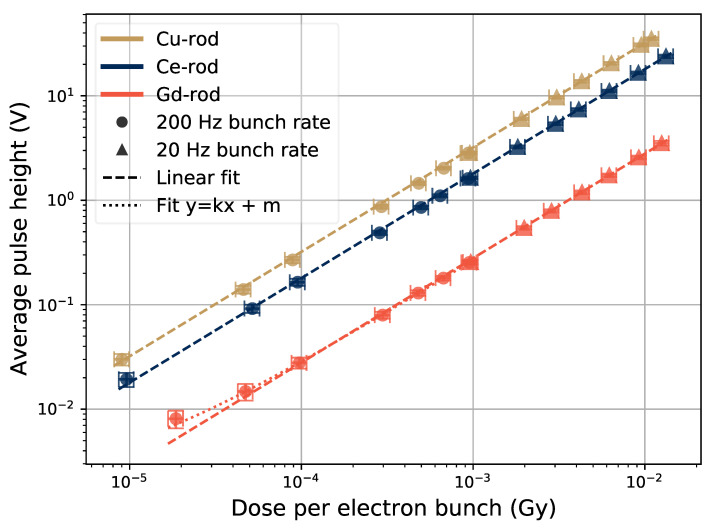
Pulse height as a function of electron bunch size in the different samples. The average pulse height is shown for electron bunch rates of 20 Hz and 200 Hz.

The relation between the height of the pulses and the size of the electron bunches is seen in Figure 7 for different sample dopants, and for dose rates up to $1.5\times 10^{-2}$ Gy/bunch. A note to keep in mind is that this dose rate corresponds to an instantaneous dose rate during a 5-μs pulse of 3 kGy/s. The data is based on 30-s irradiation runs at each electron bunch size, and the data points are located at the average pulse height (see Figure 2 and Figure 6). The error bars represent, in the *y*-axis direction, the standard deviation of the pulse height, and in the *x*-axis direction, 10% of the reported dose per pulse.

The results in Figure 7 show a linear trend over the whole tested range of bunch sizes for the Ce- and Cu-doped rods. For the Gd-doped rod, the point at $2\times 10^{-5}$ Gy/bunch is slightly above the fitted linear slope. The signal light output was smaller from this sample than the others in terms of the pulse height, and at smaller electron bunches, the signal was influenced by noise. Thus a fit to the data containing a constant factor taking into account the background noise level in the signal gives a better representation of the sample response for small electron bunches. The constant factor (*m* in the figure legend) in the dotted line was fitted to a value of $3.8\times \sigma_{noise}$, where $\sigma_{noise}=0.6$ mV was the calculated standard deviation of the signal noise in the data point at $2\times 10^{-5}$ Gy/bunch.

The actual magnitude of the data points in Figure 7 depend on many parameters. Luminescence properties of the sample is one of these parameters, but PMT gain voltage, the thickness of transport fiber, and relative orientation between the transport fibers end and PMT window are examples of parameters that will have an influence on the signal level. Thus, a test setup like this will have to be re-calibrated each time the setup is constructed. However, for a product where all relevant parameters can be kept constant or accounted for, this would not be necessary.

**Figure 8:**
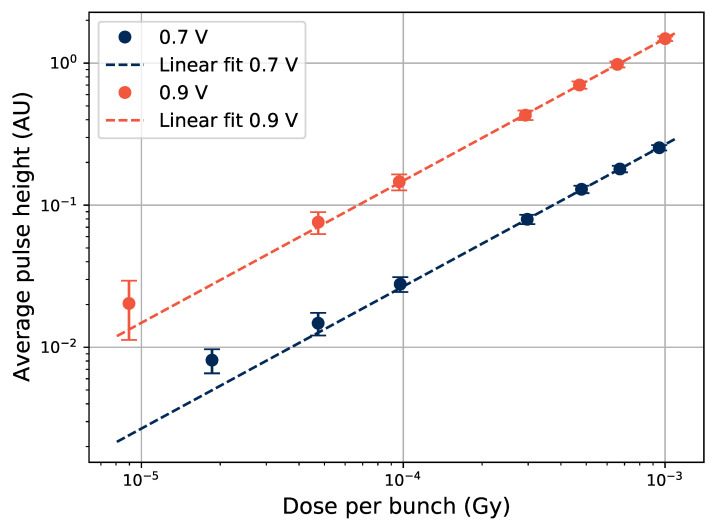
Pulse height as a function of electron bunch size in the Gd-doped sample at varying PMT gain voltages.

Adding a term to the fit to account for the background for the Gd-rod data in Figure 7, provides a better representation for the points with low dose per bunch. The effect of varying the PMT gain voltage during tests with the Gd-rod is shown in Figure 8, where data sets taken at 200-Hz bunch frequencies with PMT gains of 0.7 and 0.9 V are shown. When increasing the gain voltage from 0.7 V to 0.9 V, the signal from the low dose pulses gets represented better by the linear fit to the data. The error bars, i.e., the variations of the height of the pulses, are still large at the low dose per pulse point with 0.9 V PMT gain. Thus, the Ce- or Cu-rods, providing larger output pulses, are better suited to monitor small radiation pulses in this manner than the Gd-rod.

**Figure 9:**
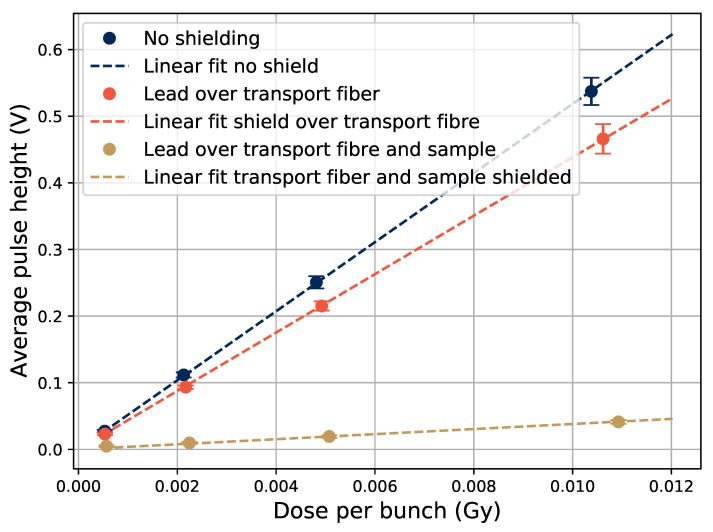
Pulse height as a function of electron bunch size in the Gd-doped sample at different shielding configurations using 5-cm thick lead bricks.

In Figure 9, three different shielding configurations were used to study where the light emission was induced in the Gd-doped sample, since no optical filter was used for this one. The *no shielding* option is the same as shown in previous figures, where the sample was in the beam center and no lead was present.

To shield different parts of the sample, 5-cm thick lead bricks were used, and when the transport fiber is put under lead, the signal decreases slightly. Čerenkov radiation is a possible source for light induced in the transport fiber, since it can be induced by 20-MeV electrons in silica. However, it has a maximum emission angle at about 45${}^{\circ }$ [25], and thus much of the induced Čerenkov light would not be transmitted, as the angle between the transport fiber direction and the beam direction was 90${}^{\circ }$, and no visible Čerenkov component could be seen in Figure 5.

**Figure 10:**
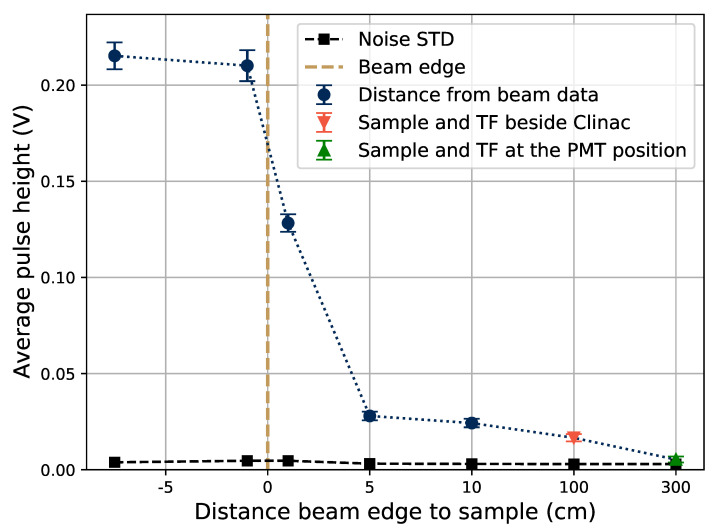
Pulse height as a function of the Gd-doped samples position relative to the edge of the electron beam. The beam spot was a square with 15-cm sides, and the point at −7.5 cm is taken in the beam center. At the point at a 300-cm distance, the sample was laying at a position beside the PMT and electronics. Electron bunches of 5 mGy/bunch at 20 Hz were used to produce this data.

When also shielding the sample, there is still a signal in the system proportional to the electron bunch size, as can be seen in Figure 9. To further examine this, the pulse height of the signal as a function of the sample position is shown in Figure 10, where the beam profile can be seen. No electron applicator was used for these tests, which would make the beam edges sharper if it was used. Even when the sample is positioned outside of the beam, as well as when the transport fiber (TF in the figure legend) is laid out beside the accelerator (about 1 m below and beside the beam area), there is some signal in the system. This shows that there is some radiation background present far from the beam, which can explain the presence of the signal in the fully shielded case in Figure 9, in which the unshielded transport fiber far from the beam has a similar position as the transport fiber laying beside the Clinac in Figure 10.

In the sample position beside the Clinac, the whole transport fiber and rod was stretched out on the floor of the irradiation hall, about 1 m from the irradiation window. The signal that is present when the sample is outside of the beam is however not likely induced in, or by, the electronics, because when the sample was located at the same position as the PMT (the green datum point in Figure 10), the signal was at the noise level. In the fully shielded case of Figure 9, some of the signal that is present will likely also come from electrons and photons penetrating the Pb shield, since the high energy electrons and the bremsstrahlung produced within the shielding material is highly penetrating.

#### 3.2.2. Variations of Pulse Area

**Figure 11:**
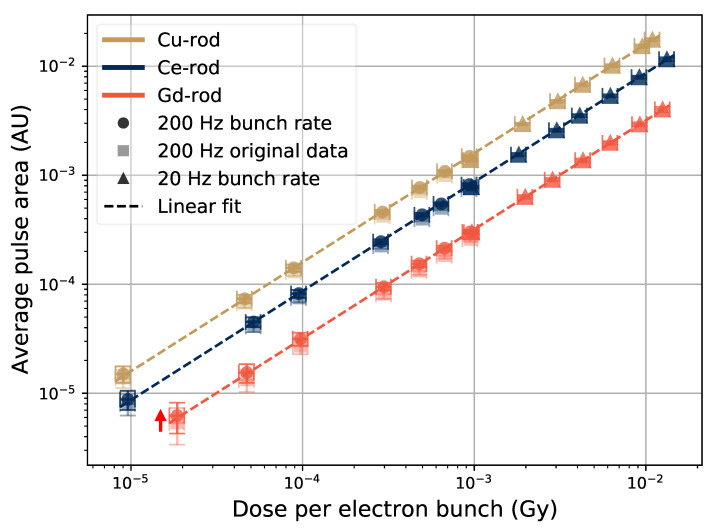
Pulse area as a function of electron bunch size in the samples. The average pulse area is shown for electron bunch rates of 20 Hz and 200 Hz.

The output pulses from the PMT have the same shape for a given sample at varying bunch sizes, and thus the area of the PMT pulses (the integrated voltage over time of the PMT pulse duration) is also proportional to the electron bunch size, since the pulse height is. The average PMT pulse area (c.f. Figure 2) as a function of electron bunch size is shown in Figure 11.

**Figure 12:**
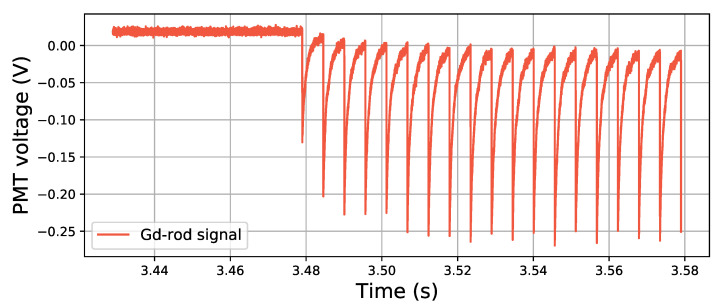
Signal from the Gd-doped sample when subjected to a pulsed beam with a frequency of 200 Hz at 1 mGy/bunch. The signal does not have time to fall back to the baseline, and the consecutive pulses are located on the tails of the previous ones.

In Figure 11, the data taken at different bunch frequencies are scaled to match with each other. There is an offset between the data at the different frequencies for the case of the pulse area, which is shown with an arrow beside the Gd-rod data in Figure 11, and faint square markers for the original 200-Hz data points. This offset is compensated with a constant factor, and it comes from the fact that the signal from the previous pulses has not yet been able to reach the baseline level when the electron bunch frequency is high. The arriving pulses are thus located on the tail of the previous pulse (illustrated in Figure 12), and this tail is then decaying, moving the baseline lower over the duration of the new pulse, thereby decreasing the effective area of the pulses.

The tails of the Gd-sample are the longest due to the long decay time of the Gd luminescence. The main decay time component of the Gd${}^{3+}$ ions luminescence is about 1.8 ms (see e.g., [26]) compared with Cu${}^{+}$ ions and Ce${}^{3+}$ ions where the decay times are on the order of 40–50-μs and 50 ns respectively [6,9,27]. The shift factor for the high-rate points was also noted to be most apparent for the Gd-doped sample. The factors used to scale the 200 Hz data to the 20 Hz data were 1.17 for the Gd-rod, and 1.10 and 1.09 for the Ce-rod and Cu-rod, respectively.

The decay times of the pulses in e.g., Figure 6 and Figure 12 differ from the decay times of the luminescence of the dopant ions. This is because they were collected with a 1-MΩ input impedance in the oscilloscope, which affects the signal pulses relaxation time. As a comparison for these samples, the fall times of the recorded pulses (here time from the peak value $V_{peak}$ to the value $V_{peak}/e$) were about 0.95 ms for the Gd-rod, 0.41 ms for the Ce-rod, and 0.40 ms for the Cu-50 sample. These are approximate values observed for the pulses behind the data in Figure 11, and are presented in Table 2 along with the decay times of the dopant ions obtained from the literature.

From the tabulated decay and fall times in Table 2, it can be seen that the Ce${}^{3+}$ and Cu${}^{+}$ ions have shorter dopant decay times than the fall time of the pulse seen in the 1-MΩ impedance oscilloscope as would be expected. Moreover, the samples containing these dopants were used with optical filters selecting the corresponding RIL emission spectral domain. Hence, the exact values of the PMT pulse fall time will depend on the total relaxation times of each of the systems, which might differ from the specific decay time of the dopant ions, along with the input impedance of the oscilloscope. For the Gd${}^{3+}$-doped rod, the dopant decay time is instead longer than the PMT pulse fall time. For the Gd-rod, no optical filter was used, and the prompt response of the sample (originating from e.g., Čerenkov light) might have a larger impact. The prompt response of the sample will quickly vanish, which for this case would make the resulting response time faster than the decay time of the dopant.

The different properties of the samples will have implications in what bunch frequencies can be resolved: A faster RIL decay makes it possible to use in a higher bunch frequency. The pulse duration could also be further adapted to the bunch frequency in the desired application, by tuning the input impedance of the readout system.

Using the pulse area instead of the pulse heights makes the noise level less significant, as uniform noise around the zero-level cancels out to zero when integrating the PMT pulse over time. Thus, no added constant factor to the linear fit, accounting for the signal noise, is needed. The error bars are however still large for signals, which are close to the noise, as seen for the Gd-rod sample at $10^{-5}$ Gy/bunch in Figure 11.

#### 3.2.3. Total Area of Traces with Many Pulses

**Figure 13:**
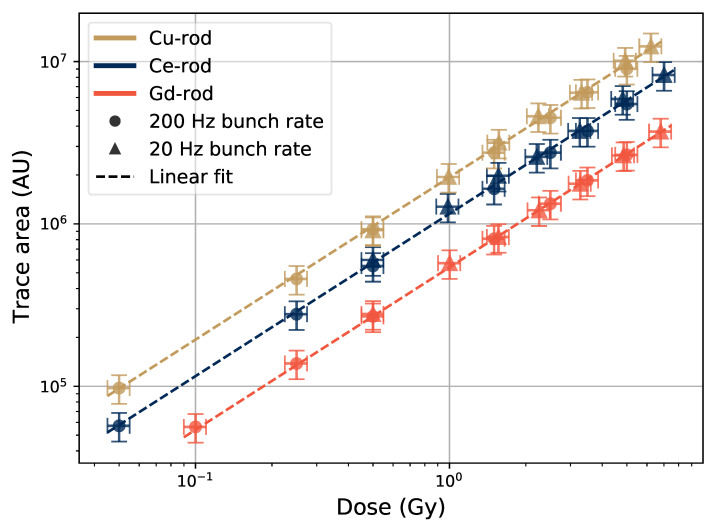
Total area of the recorded pulse traces as a function of run dose in the samples, from the same data sets as in Figure 7 and Figure 11.

Since the area of the individual pulses are proportional to the electron bunch sizes, the area of the whole recorded trace should be proportional to the dose of the full run. This is shown in Figure 13, where the *y*-axis direction error bars represent 10% of the numeric value of the trace area as a guidance value, and the *x*-axis direction error bars represent 10% of the reported dose value.

When comparing the trace area values, no correction between high and low frequency data points is needed, as the piling up of the individual pulses at high electron bunch frequencies does not change the total trace area, as seen in Figure 13, where the high and low frequency data points follow the same linear trend over the tested dose range from $5\times 10^{-2}$ Gy to about 6.5 Gy.

The noise is in Figure 13, as in Figure 11, uniform around the signal level and cancels out when the trace area is calculated (signal trace is integrated over time). For each tested rod, the data shows a good linearity between the dose absorbed by the sample and the total signal area. Compared with Figure 2, the zero level of the trace was calculated only in the beginning of each run to obtain the total trace area, and the separate pulses were not specifically taken into account.

## 4. Conclusions

In this study, the RIL response of doped sol-gel silica glass samples to a pulsed electron Clinac beam was investigated for the first time to the best of our knowledge. In effect, studies addressing the RIL from luminescent glassy materials in a fibered system to probe electron beams are scarce. Moreover, the doped sol-gel silica used here is quite different from materials appearing in references [12,13,14,15,16,17]. The response of the tested samples was also studied in a pulse-by-pulse manner for each impinging electron bunch. It was found that the height and area of the output PMT pulses were proportional to the dose of the impinging electron bunches in the range $10^{-5}$ Gy/bunch—$1.5\times 10^{-2}$ Gy/bunch. The total integrated trace areas of the irradiation runs were also found to be proportional to the dose from the electron beam during the runs. Based on these results, it is concluded that these samples have strong potential to be used for radiation monitoring of electron Clinac beams.

These observations were true for all tested samples. However, choosing an appropriate sample with properties matching the desired beam parameters is necessary. The sample needs to have a high enough light output so the signal from the bunches is visible. In this case, parameters regarding sample geometry and doping are important to consider, as well as signal amplification such as the PMT gain. Further care should be taken at high bunch frequencies of irradiation, so that the RIL in the sample can fully decay between consecutive bunches. Alternatively, this could be compensated if necessary by applying an offset factor depending on the bunch frequency and sample that is used.

Part of the induced signal was found to originate within the transport fibers, and not from the RIL of the dopant ions in the samples. A linear agreement between the induced signal and the dose of the electron bunches was still observed, but an important consideration is to keep a controlled transport fiber orientation relative to the beam, to minimize variations in, for example, induced Čerenkov radiation between runs.

In a radiation environment from a Clinac in use for radiotherapy, the variation of electron bunch sizes would be much smaller than the range studied in this paper. The variations of the bunch sizes in a Clinac in operation could originate from varying depth in a target material or possible variations between different accelerator models. These variations would occur within the central region of the studied range, where the tested samples would be well suited to monitor the dose deposited by each electron bunch, or the total dose during a run with many consecutive bunches.

## Figures and Tables

**Table 1:** Summary of tested samples.

Sample	Dopant	Dopant Concentration (wt%)
Ce-rod	Ce${}^{3+}$	0.07
Cu-rod	Cu${}^{+}$	0.07
Gd-rod	Gd${}^{3+}$	0.1

**Table 2:** Time structure of the pulses from the samples.

Sample	Dopant	Dopant Decay Time (s)	PMT Pulse Fall Time to $V_{\mathrm{peak}}/e$ (s)
Ce-rod	Ce${}^{3+}$	$50\times 10^{-9}$ [6,27]	$4.1\times 10^{-4}$
Cu-rod	Cu${}^{+}$	40–50 $\times 10^{-6}$ [9]	$4.0\times 10^{-4}$
Gd-rod	Gd${}^{3+}$	$1.8\times 10^{-3}$ [26]	$9.5\times 10^{-4}$

## Data Availability

The data that were analyzed to produce this publication are available through the JYX database (https://jyx.jyu.fi/handle/123456789/78578, accessed on 7 November 2021).
